# Challenges for the implementation of World Health Organization guidelines for acute stress, PTSD, and bereavement: a qualitative study in Uganda

**DOI:** 10.1186/s13012-016-0400-z

**Published:** 2016-03-15

**Authors:** Jeremy C. Kane, Alex Adaku, Juliet Nakku, Raymond Odokonyero, James Okello, Seggane Musisi, Jura Augustinavicius, M. Claire Greene, Steve Alderman, Wietse A. Tol

**Affiliations:** 1 Department of Mental Health, Johns Hopkins Bloomberg School of Public Health, 624 North Broadway, Baltimore, MD 21205 USA; 2 Peter C. Alderman Foundation Uganda, Arua, Uganda; 3 Peter C. Alderman Foundation Uganda, Kampala, Uganda; 4 Department of Psychiatry, Makerere University, Kampala, Uganda; 5 Peter C. Alderman Foundation Uganda, Gulu, Uganda; 6 Peter C. Alderman Foundation, Bedford, NY USA

**Keywords:** World Health Organization, Clinical guideline implementation, Post-traumatic stress disorder, Acute stress, Bereavement, Uganda, Low- and middle-income country

## Abstract

**Background:**

In 2013, the World Health Organization (WHO) published new guidelines for the management of conditions specifically related to stress, including symptoms of acute stress, bereavement, and post-traumatic stress disorder (PTSD). It is important to evaluate potential challenges for the implementation of these guidelines in low-resource settings, however, there is a dearth of research in this area. The current qualitative study aimed to assess perspectives on the feasibility and acceptability of the new guidelines in four clinics that provide mental health services in post-conflict northern Uganda.

**Methods:**

In-depth interviews were conducted with 19 mental health-care providers and program developers in northern Uganda to address three major research objectives: (1) describe the current standard practices and guidelines used for treating conditions related to stress in Uganda; (2) identify barriers and challenges associated with implementing the new WHO guidelines; and (3) identify and describe potential strategies for overcoming these barriers and challenges. An emergent thematic analysis was used to develop a coding scheme for the transcribed interviews.

**Results:**

Practices for managing conditions related to stress included group psychological interventions, psychoeducation, and medication for clients with severe signs and symptoms. Several themes were identified from the interviews on barriers to guideline implementation. These included (1) a lack of trained and qualified mental health professionals to deliver WHO-recommended psychological interventions; (2) a perception that psychological interventions developed in high-income countries would not be culturally adaptable in Uganda; and (3) reluctance about blanket statements regarding medication for the management of acute stress symptoms and PTSD. Identified strategies for overcoming these barriers included (1) training and capacity building for current mental health staff; (2) a stepped care approach to mental health services; and (3) cultural modification of psychological interventions to improve treatment acceptability by clients.

**Conclusions:**

Guidelines were viewed positively by mental health professionals in Uganda, but barriers to implementation were expressed. Recommendations for implementation include (1) strengthening knowledge on effectiveness of existing cultural practices for improving mental health; (2) improving supervision capacity of current mental health staff to address shortage in human resources; and (3) increasing awareness of help-seeking clients on the potential effectiveness of psychological vs. pharmacological interventions.

**Electronic supplementary material:**

The online version of this article (doi:10.1186/s13012-016-0400-z) contains supplementary material, which is available to authorized users.

## Background

Exposure to war and conflict is associated with psychological distress, including acute stress reactions and bereavement, in both child and adult populations [1–4]. In a meta-analysis of mental health outcomes associated with trauma among populations affected by conflict or displacement, Steel and colleagues calculated an adjusted post-traumatic stress disorder (PTSD) prevalence of 15.4 % across the most rigorous studies [5]. PTSD has been the most commonly studied mental disorder in these contexts, although there is broad recognition that a range of mental disorders are of public health importance in conflict-affected settings. This range of mental disorders includes both conditions specifically associated with stress, that is, those requiring exposure to major stressors, as well as conditions that may also occur without exposure to major stressors (e.g., depression, anxiety disorders, substance use, suicidality) [6]. Exposure to armed conflict is of particular concern in low- and middle-income countries (LMIC), where the majority of today’s wars and conflicts are taking place [7].

In Uganda, a brutal civil war was fought for more than 20 years between a rebel faction, the Lord’s Resistance Army (LRA), and government forces [8, 9]. Hostilities formally came to an end in early 2008; however, the effects of the war have persisted [10]. Although the entire country suffered, the war particularly impacted the northern and eastern geographic regions of Uganda, resulting in thousands of deaths, over 1.5 million displaced persons, and 30,000 child abductions by the LRA including many cases of children forced into combat roles [8, 9, 11]. Exposure to conflict-related events in Uganda has been linked to an increased risk for PTSD [8, 12]. Studies have also suggested that in post-conflict settings, stressors such as stigma and domestic violence are associated with negative psychological consequences [8, 13].

Current guidelines for the treatment of PTSD and other mental health problems specifically related to stress in LMIC, such as Uganda, may be inappropriate because they are often developed for use in highly resourced settings [14] and may have conflicting messages, depending on the audience to whom guidelines are addressed [15]. In response to the limitations in current guidelines for mental health service in LMIC overall, in 2009, the World Health Organization (WHO) launched the Mental Health Gap Action Programme (mhGAP), an effort to increase accessibility to evidence-based treatments for mental health problems that has been used in over 50 countries [16, 17]. Subsequently, the WHO published an intervention guide (mhGAP-IG) based on the best evidence-based practices. The mhGAP-IG was an effort to expand and strengthen mental health services in LMIC and to provide direction in the management of mental, neurological, and substance use (MNS) problems in nonspecialized health settings [17, 18]. Several research consortia are evaluating the application of the mhGAP guidelines in multiple LMIC, including in Uganda [19–21].

Although the mhGAP intervention guide covered a wide range of MNS problems, it did not specifically address evidence-based practices for the management of conditions specifically related to stress (e.g., PTSD, bereavement, and acute stress). Therefore, in 2013, the World Health Organization (WHO) issued *Guidelines for the Management of Conditions Specifically Related to Stress* as an additional module to the existing guidelines [4]. These new recommendations and intervention guide are available online and will be incorporated in a revised version of the overall mhGAP package. In addition, WHO and UNHCR have collaborated to develop a mhGAP Humanitarian Intervention Guide (mhGAP-HIG), which includes the recommendations on conditions specifically related to stress [22]. This document has been disseminated through inter-agency collaboration platforms as well as in the peer-reviewed literature [23]. As far as we are aware, however, further systematic dissemination of the guidelines for conditions specifically related to stress has not been conducted, and we are not aware of published accounts of barriers and facilitators to systematic implementation of these specific guidelines within the mhGAP package.

Recently, Tol and colleagues (2014) discussed key challenges anticipated in implementing these guidelines in LMIC. Chief among these anticipated challenges was the lack of resources in LMIC available or devoted to mental health services, including a lack of trained professionals in specialized mental health care (e.g., trauma-focused cognitive behavioral therapy). Other challenges anticipated included a potential disinclination of providers to change their current practices (if those practices stood in contrast to the new WHO guidelines) and the degree to which the guidelines may (or may not) generalize to LMIC settings and different cultural contexts. In conclusion, the authors recommended that investigations, including qualitative research studies, were warranted to assess barriers and facilitators of dissemination and implementation of guidelines in these settings and gain insight into how evidence-based interventions may best be adapted for diverse LMIC settings [17].

The anticipated challenges relating to integration of guidelines into current practices, resource availability, and generalizability resemble barriers to implementation of mental health guidelines that have been identified in high-income settings [24, 25]. Studies from high-income countries have indicated that published guidelines for both acute and chronic conditions [26, 27], including mental health problems [28, 29], had low compliance and that most strategies for implementing guidelines are minimally effective or typically not applied in real-world settings. Effecting change in clinical practice in accordance with guideline recommendations typically extends beyond the guidelines themselves and requires multifactorial and multi-level strategies to address these ubiquitous barriers to implementation [30]. Dissemination and implementation researchers have described this process as knowledge translation, which is defined as bridging the gap between research and policy or practice [31].

Knowledge translation exists on a continuum beginning with evidence synthesis and ending with uptake and evaluation of recommendations [31, 32]. In mental health research in high-income countries, the majority of knowledge translation, dissemination, and implementation efforts have focused on changing practices and knowledge at the provider level and, while evidence has been mixed, some strategies have shown to be capable of increasing uptake and utilization of recommended practices [33]. As summarized by Goldner et al. (2011) in a scoping review of knowledge translation in mental health [33], “active” translation strategies for mental health guidelines, such as tailored in-depth educational activities, access to expert consultants, and resource availability through webinars, listservs and online forums were more effective than “passive” strategies, in which guidelines were disseminated to practitioners (e.g., through mailing) but no additional training or direction was provided. Additionally, “integrated” implementation efforts that included input from stakeholders across various levels (clients, providers, policy-makers, researchers, and community groups) from the outset were also found to result in successful guideline implementation [34].

Research from LMICs on guideline implementation is scarce [35]. In this qualitative case study, we examine perspectives on challenges to WHO guideline implementation and potential ways to overcome these in Uganda. Specifically, we interview mental health-care staff members in northern Uganda to identify perspectives on:

1. The most commonly encountered types of conditions related to stress and the current practices for managing these conditions.
2. Feasibility and acceptability of the new WHO guidelines and perceptions of barriers and challenges to their implementation.
3. Potential means to overcome challenges and barriers to the implementation of new WHO guidelines.

## Methods

### Study setting

In an effort to address the high prevalence of conditions related to stress and other mental health concerns in northern Uganda, and due to a shortage of mental health services, in 2005, the Ugandan Ministry of Health and the Makerere University Department of Psychiatry entered into a public-private partnership with the Peter C. Alderman Foundation (PCAF) [36]. PCAF, based in New York, was established to honor Peter C. Alderman, who was killed in the terrorist attacks on September 11, 2001 [37]. The partnership is aimed at building capacity of the Ministry of Health and other key stakeholders to deliver culturally appropriate, accessible, and evidence-based mental health supports for conflict-affected populations. As part of this mission, PCAF in Uganda is interested in assessing the locally perceived relevance and feasibility of implementing the new WHO guidelines.

The partnership has established mental health teams in four districts in northern and eastern Uganda: Gulu, Kitgum, Arua, and Soroti [36]. Mental health services consist of static clinics and ongoing community mental health efforts. The static clinics are all housed within hospitals in each district. Clinic staff include psychiatric clinical officers, psychiatric nurses, social workers, and counselors/psychologists. This four-person team operates under the supervision of a staff psychiatrist. Community mental health interventions consist of liaison with lower level health centers and their community health workers (the Village Health Team), bi-weekly outreach to rural areas by the static clinic members (including to refugee settlements), and continuous presence through community social workers. Treatment options for the broad range of mental health concerns seen in the static clinic and outreaches include both medication (e.g., antipsychotics, antidepressants, benzodiazepines, anticonvulsants) and psychosocial interventions (e.g., group counseling, individual counseling, family therapy, traditional/faith healing, home visits) [36]. Through these activities, the PCAF teams treat approximately 1069 clients per month.

### Participants and procedure

Study participants were mental health-care professionals (psychiatrists, psychiatric clinical officers, counselors, psychiatric nurses, and social workers) at the four sites in northern Uganda. We attempted to interview each of the five members of the mental health team at all clinics, although this was not possible at all sites due to staff members being away on leave during the study period. We also interviewed senior-level psychiatrists and other officials who are mental health focal points in Uganda and primarily based in the capital city, Kampala.

Interviews were conducted in a private room within the mental health clinics at the four northern Uganda sites. For the interviews with participants in Kampala, interviews were conducted in private offices. All interviews were digitally recorded and then transcribed onto a study laptop at which time the digital recording was destroyed. All transcribed interviews were de-identified such that names and locations were removed. Interviews lasted for approximately 60 to 90 min. All interviews were conducted in English.

The interview guide included questions in three clusters, one for each of the three research objectives. The first cluster of questions focused on the common types of problems that were treated by the mental health team and the current practices for managing those problems.

The second cluster focused on the WHO guidelines themselves. PCAF staff in Uganda have been trained and are supervised in current methods for managing mental disorders relevant for conflict-affected populations. Most participants had heard of the WHO mhGAP guidelines, but except for one of the psychiatrists in the sample, PCAF staff had not been trained specifically in using the WHO mhGAP intervention guide and none had been trained in the new module for conditions specifically related to stress. At the first site, we therefore formally presented these guidelines to the entire team before conducting the individual interviews. However, we noticed that some participants relied on this formal presentation when expressing opinions on the first set of questions. Therefore in all subsequent sites, we did not have a formal introduction of the guidelines, but rather the interviewer introduced the guidelines informally (see Table 1) to the respondent during private interviews as part of this second cluster of questions. After introducing each WHO guideline, the interviewer would prompt the participant for his or her feedback and probe for challenges or barriers associated with implementing that particular guideline in the participant’s clinic. We found that the informal presentation of the guidelines resulted in participants providing a greater number of perceived barriers and challenges and a greater breadth of responses.

**Table 1:** Summary of World Health Organization mhGAP guidelines for management of conditions related to stress

Condition	Intervention	Client	Recommendation
Acute stress symptoms	Psychological	Adults	CBT-T recommended. No recommendation for EMDR, “stand-alone” problem-solving counseling, or psychoeducation. Stress management may be used if CBT not available along with PFA.
		Children	No specific recommendation^a^. PFA may be considered.
	Pharmacological	Adults	Benzodiazepines and antidepressants should not be offered.
		Children	Benzodiazepines and antidepressants should not be offered.
Secondary acute insomnia	Psychological	Adults	Relaxation technique and sleep hygiene advice should be provided through existing cultural practices recommended.
		Children	No specific recommendation^a^. Relaxation techniques and sleep hygiene advice may be safe and effective in adolescents.
	Pharmacological	Adults	Benzodiazepines should not be offered apart from exceptional cases in which short-term use (3–7) days may be considered.
		Children	Benzodiazepines and antidepressants should not be offered.
Secondary nonorganic enuresis	Psychological	Children	Psychoeducation and skills training for parents recommended.
Dissociative disorders	Psychological	Adults	No specific recommendation^a^. PFA and the use of culturally appropriate interventions that are not harmful may be considered.
		Children	
Hyperventilation	Psychological	Adults	No specific recommendation for rebreathing into a paper bag^a^.
		Children	Rebreathing into paper bag should not be considered.
PTSD	Psychological	Adults	Group or individual CBT-T, EMDR, and stress management are recommended.
		Children	Group or individual CBT-T and EMDR are recommended. Stress management may be beneficial.
	Pharmacological	Adults	SSRIs and TCAs should not be offered as first-line treatment but may be considered if psychological interventions are not available or if there is co-occurring depression.
		Children	Antidepressants should not be used.
Bereavement	Psychological	Adults	Structured psychological interventions should not be universally offered. General principles of care (providing and mobilizing social support, encouraging culturally appropriate mourning) should be used.
		Children	
	Pharmacological	Adults	Benzodiazepines should not be offered for individuals who do not meet criteria for a mental disorder.
		Children	

*Abbreviations*: *PFA* psychological first aid, *EMDR* eye movement desensitization and reprocessing, *CBT-T* cognitive behavioral therapy with trauma focus, *SSRI* selective serotonin reuptake inhibitor, *TCA* tricyclic antidepressant

^a^No recommendation could be given due to insufficient evidence

The third cluster of questions focused on methods or strategies for overcoming barriers or challenges mentioned in the second cluster. If the participant did not foresee any barriers or challenges, the interviewer asked about challenges that were mentioned by other participants.

### WHO guidelines

The WHO guidelines were each reviewed with the participants during the individual interviews. The guidelines cover the following: (a) symptoms of acute stress (acute traumatic stress symptoms, secondary acute insomnia, secondary nonorganic enuresis, dissociative disorders, hyperventilation); (b) PTSD; and (c) bereavement. The recommendations for each are further sub-divided by age (adults and children) and management option (psychological and pharmacological) [4]. A summary of the guidelines, similar to what was shared with the study participants, is displayed in Table 1.

### Qualitative data analysis

Based on the approach to grounded theory by Charmaz (2006), our methodology recognized the role of the investigator in the research process and that the findings from this study are constructions of reality [38]. Codes and categories for understanding the providers’ perceptions on the implementation of the WHO guidelines were constructed from the data rather than from preexisting theories. We used a version of a constant comparative data analysis method, whereby we developed themes in early interviews and then compared information gathered from subsequent interviews to these themes, updating them when necessary. Coding was done manually using an in vivo method to explore themes that we identified in the data, where concepts were coded based on language provided by the study participants [39, 40]. We also explored whether themes varied across clinic sites and provider type.

The analysis was conducted by three researchers (authors JK, MCG, and JA). All three researchers first completed an independent analysis. This was done by reading all of the transcripts line-by-line and coding themes the researchers identified in the data (following methods described above) within each of the three a priori study objectives: (1) commonly treated mental health problems and current methods for managing these problems; (2) barriers and challenges to implementation of WHO guidelines; and (3) strategies and facilitators for implementation of WHO guidelines. Each researcher provided names, definitions, and accompanying illustrative quotes for each theme that they identified within each objective. Following the independent coding, all three analysts met to discuss findings and reconcile differences until consensus was reached on the final themes and which quotes best represented these themes. Illustrating examples of the themes are presented through (de-identified) participant quotes that were thought to be representative of the theme. The themes, definition of themes, and examples are presented in supplementary tables (as Additional files 1, 2, 3, and 4) for each research objective as per Valentine et al. (2014) [40].

### Ethics and consent

The study was designated as exempt by the Johns Hopkins Bloomberg School of Public Health Institutional Review Board and was approved by the Makerere University Ethical Review Board in Uganda. Administrative permission to carry out the study was obtained at each of the hospitals where the static clinics were located. Informed consent was obtained from the study participants after explaining the study including risks and benefits.

## Results

Participants were 19 mental health professionals in Uganda. This included 17 members of the PCAF mental health teams including three psychiatrists, four psychosocial counselors, four social workers, three psychiatric clinical officers, and three psychiatric nurses. Interviews with three team members (one psychiatrist, one psychiatric clinical officer, and one nurse) could not be completed because those individuals were away on leave during the study period. Additionally, we interviewed two officials involved with mental health programming for non-governmental organizations. Several themes were identified from each of the three research objectives. There were no substantive differences in responses across sites (clinics). A difference across provider type was noted for one theme (use of medication, described in objective 1, part vi). Themes are described in detail below and summarized in Tables S2, S3, S4, and S5 (included as Additional files 1, 2, 3, and 4).

### Objective 1: identify the common types of conditions related to stress that are treated in this setting and the types of methods used for managing these conditions

#### Conflict-related trauma continues to affect mental health, but ongoing adversities are important

Most of the participants reported that war-related hardships were the most commonly reported event type associated with ill mental health. They explained that even though the civil war in northern Uganda officially ended almost 10 years ago, the burden of ill mental health associated with the conflict experienced by many Ugandans has persisted. This is due in part, as one participant explained, to the fact that many conflict-affected persons have not received mental health support in the ensuing years. Furthermore, the ongoing conflict in South Sudan has resulted in an influx of refugees to northern Uganda. Participants reported that there was a need for services for conditions related to stress among this population.

Ongoing post-conflict stressors such as lost relatives, hardships associated with physical injury, gender-based violence and changed gender roles, and loss of income and land were also described as important factors influencing mental health. For example, recent adversities such as domestic violence and “land wrangles,” disputes over land often resulting in violence, were consistently mentioned as post-war events that were reported by a number of clients seeking services. Many of the providers described these more recent stressors as “triggering” events that led to recall of earlier adversity experienced during the war itself. This was thought to be particularly problematic among those clients who had not previously received any sort of mental health care for conflict-related mental health concerns.

#### PTSD is commonly identified but often comorbid with other mental health conditions

Most of the participants reported that PTSD symptoms were very commonly seen in their work; however, these statements were often qualified by suggestions that clients typically had more than one condition at a time. Among these co-occurring mental health problems, PTSD and depression were the most often cited (referred to by some participants as “complex PTSD”). Alcohol and substance abuse were also mentioned as occurring frequently among those who present with PTSD symptoms. One participant concluded, “comorbidity is the norm rather than the exception.”

#### Conditions not related to stress are commonly reported in clinics

Despite the fact that the participants we interviewed were commonly focused on mental health in the context of armed conflict, many reported providing care for clients with symptoms that were not related to major traumatic stressors. Epilepsy was frequently mentioned as a condition that was referred to their clinics. Alcohol and substance use (not trauma-related) was also commonly reported in addition to fewer cases of anxiety and psychotic disorders.

#### Assessment is done in a holistic manner

The word “holistic” was mentioned by a number of participants with regard to the manner in which clients are assessed and referred for appropriate treatment. Participants described an assessment in which the client’s physical health, emotional, and social well-being were all evaluated. Based on this comprehensive evaluation, a client was said to be referred to one or more specialists, depending on the presenting signs and symptoms. For example, a person with social or domestic problems would be referred to the social worker and a person with symptoms associated with major stressors would be referred to the psychosocial counselor.

#### Psychoeducation is a significant component of management

Psychoeducation was mentioned by almost all of the participants as an important component of treatment. They described a “health talk” which was given each morning to clients in the waiting room. During these talks, a member of the clinic team would describe common signs and symptoms of conditions specifically related to stress, the etiology behind these conditions, and options for self-management and treatment. Some participants went further to say that it was an important part of the management. As explained by one of the psychiatrists:

A lot of people know they have gone through trauma but they cannot link it to the symptoms or the mental health problems that they are presenting with. What we are doing in the clinic most of the time is providing psychoeducation to link the trauma to the symptoms and hopefully it helps both: dealing with the trauma and also relieving the signs and symptoms of trauma.

#### Medication is frequently used in the treatment for conditions specifically related to stress among clients who are considered to have severe signs and symptoms

Many of the participants expressed the need to use medication when treating clients with severe symptoms. For many participants, medication was a natural prerequisite to beginning psychological interventions. This was thought to be necessary for complex cases of PTSD (e.g., PTSD comorbid with depression) but also for PTSD alone if the symptoms were thought to be severe. Many believed that with symptoms that were “high,” psychological interventions would be either ineffective or impossible to implement as a stand-alone treatment. There were differences across provider type, however, with this practice more often supported by psychiatrists as compared to counselors and social workers.

#### Group therapy is the preferred psychological intervention

Participants expressed several reasons why they believed that group psychotherapy was advantageous to individual counseling. Chief among these reasons was the efficiency: large patient populations and a small number of qualified counselors made group therapy a much more feasible option than individual counseling. Many also believed that a group method was more culturally appropriate and therefore more effective than individual therapy. Clients with similar presentations can be grouped together, and as one participant put it, “they can encourage each other and get [learn] from one another how one is coping up.”

#### Clinic-based populations may have different treatment needs than outreach/refugee settlement populations

Participants emphasized that the types of mental health problems managed in their static clinic settings were often different than the types of problems experienced in refugee settlement and other rural outreach sites. Post-traumatic stress symptoms may be more common among clients in the outreach sites, possibly a reflection of the recent surge in the South Sudanese refugee population. The management of conditions is also complicated in the refugee resettlement sites, in which access to services are low and social situations are often worse than in the urban and peri-urban areas where the clinics are located.

I want to give a scenario, there was a colleague, she was a wife to some army officer in Congo, and the husband was killed and she was here in the camp, they actually killed the husband and she was raped, and the husband was killed so she just could not sleep. And she was sleeping on carpets. So we were trying to bring this sleep hygiene. There is a lot of difference between people in the camp and in the nicer area in town here. So it is hard.

#### Treatment strategies are perceived to have a great number of challenges

In general, the participants believed that their current methods for managing conditions related to stress were effective. They also expressed, however, a number of challenges that either reduce the effectiveness of the interventions or render them extremely difficult to perform. These challenges included (1) a shortage of trained and qualified staff, especially for specialized interventions resulting in infrequent or irregularly scheduled treatment sessions; (2) the subsequent necessity for multitasking that goes along with the shortage; (3) insufficiencies in their own training and ability to perform various types of interventions; (4) managing their own stress and self-care; and (5) a lack of resources, such as a consistent medication supply for clients with severe symptoms.

### Objective 2: identify perceptions of barriers and challenges to implementing WHO guidelines

#### Need for additional training to administer the recommended psychological interventions

The providers believed that cognitive behavioral therapy with a trauma focus (CBT-T), one of the recommended psychological interventions for PTSD, was an effective intervention. Many expressed, however, that implementing CBT-T on a wide basis would be a challenge due to a dearth of qualified staff to administer the therapy. Participants also expressed the need for training in other psychological interventions endorsed by the WHO guidelines, including eye movement desensitization and reprocessing (EMDR) and psychological first aid. One participant asked rhetorically, “What if there is no expertise to do CBT and EMDR?” In this context, a number of participants questioned the effectiveness of short-term classroom-based trainings that have been popular in Uganda:

Of course, you cannot expect to transform someone from a complete novice in mental health to an expert in five days. Even teaching somebody how to use a protocol. They have to remind themselves which elements are important before I can refer to a protocol…Whether classroom work translates into practical changes is a big issue.

#### The patient/provider ratio is too high

Many participants stated the need for more “manpower” in order to effectively and appropriately deliver the interventions endorsed in the WHO guidelines. This was described as a function of both a small number of trained mental health professionals as well as a large patient population in need of care. Some participants expressed the lack of personnel as the most significant obstacle for implementation of the guidelines in their clinic and that this could lead to provider burn-out.

So when you see people with those symptoms and you told them you are not giving them drugs but just individual counseling sessions, you know you are going to take time. Not less than 30 minutes per patient. So how many patients are you going to see in a day if they are 75 and you are 3 people? Can you?

#### Providing regular and frequent treatment to clients

Difficulty in consistently and frequently reaching clients who are in need of regular care (e.g., weekly therapy sessions) was mentioned by many participants as a significant barrier to providing quality psychological interventions. The challenge is especially acute for clients in Uganda who reside in rural areas or refugee settlements. The interruption of counseling sessions was thought to diminish the effectiveness of the treatment and also extend the necessitated course of management resulting in other clients being forced to wait even longer for care. Counselors described having to repeat entire sessions because clients had forgotten most or all of the ground that had been covered in previous ones. Some noted that providing medication for extended periods was logistically less challenging than providing psychological interventions on a regular basis for a similar time period.

#### A preference and expectation for pharmacological treatment among help-seeking clients

A common sentiment from the participants in the study was the challenge provided by clients’ expectation for receiving medication. How, they asked, are we supposed to use only psychological interventions (in the case of acute stress and bereavement per the WHO guidelines), when clients demand medication upon arrival at the clinic? Participants indicated that offering only psychological management would result in one of two scenarios. In the first, a client would simply leave the clinic in hopes of finding a doctor elsewhere to prescribe medication. In the second scenario, the client would stay but not be engaged in the psychological intervention or believe in its effectiveness, therefore diminishing its ability to help alleviate his/her symptoms. This often led to clients dropping out of treatment altogether. A nurse succinctly explained, “So when they come here and you don’t give them medication [snaps fingers], that is the end.”

#### Providers believe that medication is necessary for the management of severe symptoms

Clinicians often believed that it was appropriate to prescribe medication for clients with severe symptoms, and so, the use of medicine by providers was also viewed as a challenge to guideline implementation. Many of the clinicians in the study suggested that they did not feel comfortable initiating CBT-T or another psychological intervention without first ensuring symptom reduction through psychopharmacological management for clients with severe symptoms. Although they supported the use of psychological interventions, they did not want to be restricted from using medication in certain cases.

#### Perceptions that cultural acceptability of psychological interventions may be low

A smaller group of participants believed that some of the interventions suggested in the WHO guidelines were unlikely to be effective in Uganda due to cultural differences. A number of participants focused specifically on EMDR and its inappropriateness for typical rural Ugandans. Some discussed cultural issues more generally as challenges to psychological interventions. For example, traditional beliefs about the cause of mental health problems can pose a challenge to providers with regard to treatment acceptability by clients. As described by one participant:

Sometimes when clients come with their caregivers, there are cultural perspectives that have been going on all this while. They will say ‘this person has been bewitched’…These are the challenges we face.

#### Poor social, health, and economic situations limit the long-term impact of mental health interventions

Many participants expressed frustration that the psychological interventions recommended by the WHO guidelines could be effective, but their ability to provide lasting effects was limited due to poor social, health, and economic situations for many of the clients. Domestic violence was frequently mentioned as a social issue that could cause clients to relapse. Clients often have serious physical health problems with low access to care or difficulty finding work. One participant took exception the guideline for providing “sleep hygiene advice” to clients with insomnia: “Because if you look at this in the camp, the shelters they are sleeping in, the beds are not there; some people are sleeping on carpets so they will not sleep.” Another described clients as improving following psychological interventions but feeling unfulfilled. Yes, the symptoms may have reduced in the short term but, as he puts it, “I lost my cattle, my property, what next?”

#### Structural barriers to implementation

Participants indicated that there were a number of structural barriers that impede their ability to conduct the interventions recommended in the guidelines. These included resource limitations, including nonhuman resource limitations such as medication availability, management and communication problems, relationships with local community leaders, and transport issues, such as difficulty in reaching clinics among clients who live far away.

### Objective 3: identify strategies for overcoming barriers to guideline implementation

#### Training and capacity building for current mental health staff

Nearly all of the participants believed that additional training in specific psychological interventions would be necessary to implement the new guidelines effectively, particularly, they emphasized, because the guidelines emphasized the use of CBT-T and EMDR. One participant explained that there was a “big gap in psychotherapy” and “few counselors” and that this gap would need to be closed in order to be able to provide CBT-T and EMDR to those who needed these types of therapies. There was no suggestion among participants, however, on the structure or length that such a training would take in order for it to be successful.

#### Additional staff to reduce the patient/provider ratio

To combat the enormous ratio of providers to clients, several participants believed that more trained staff would be the difference in whether the guidelines could be implemented or not. This included not only highly trained mental health professionals, such as psychiatrists and psychologists, but also social workers and community mental health workers. Important here, one noted, was both the qualification and gender of the new staff members. For example, in her site, there was only one female community mobilizer who was able to assist her in reaching out to female clients. She stated, “So for me, to have another female human resource, community mobilizer, that one would help.”

#### Resource allocation and task sharing

Many participants believed that a large number of additional specialized staff would not be necessary if they were to use an approach that consisted of resource allocation and task sharing, similar to a stepped care approach. They described a scenario in which community members currently in place (some referred to as village health teams and some as community mobilizers) could be trained in basic counseling skills. These community members could then conduct basic counseling sessions with clients on days and weeks when the mental health team could not be physically present at a rural outreach site or refugee camp:

A stepped care: that is what I am thinking. So you have very few people who would need EMDR, psychiatric care. So for every program in my view you have to have those steps. And so you have family, community, provider levels, and then you have more advanced care, which has fewer people.

#### Allowing for guideline flexibility when possible

Some participants argued that the guidelines could be useful, but primarily as a framework. The ability to treat clients on a case-by-case basis and the ability to prescribe medication when they believed that it was clinically appropriate were points on which some providers were unwilling to concede their specialized training to guidelines. One participant summed up his opinion on the guidelines with “you can’t put a hard and fast rule.” In other words, they argued that the guidelines could be effectively implemented if they had room within those guidelines to operate, to enact their own clinical judgment.

#### Cultural adaptation of psychological interventions

In order for interventions such as EMDR and even CBT-T to be effective in Uganda, many participants believed that cultural adaptation would be necessary. They thought that in order to have both providers and clients buy-in to the guidelines, the interventions would need to make sense for the average Ugandan. One participant believed that EMDR could be quite effective in Uganda, and that if it did not, it would be due to “poor planning.”

#### Additional psychoeducation to clients

Psychoeducation is already an integral part of clinical care for many of the participants, but a number of them believed that additional educational activities were warranted in order to achieve the buy-in necessary for the guidelines to be successful. Some suggested community sensitization meetings in order to expand the reach of the current “health talks” and to promote self-management. One participant believed simply, “I think what we just need to do is to try to continue sensitizing about how psychotherapy works.”

#### Group therapy delivery of psychological interventions

Participants agreed that psychological interventions recommended by the guidelines were appropriate treatment options for conditions related to stress. They recognized, however, that individual treatment approaches for a large number of clients in need would be impossible given the small number of trained providers. Providing psychological intervention in group format (notably, a current practice in PCAF clinics) was therefore mentioned by several participants as a strategy for overcoming the high patient to provider ratio.

#### Prioritizing staff psychosocial well-being

Participants reported that a direct outcome of the high patient to provider ratio was increased stress and possible burn-out among mental health professions. Participants believe that this could eventually lead to providers being less effective in providing care and high staff turnover. They suggested that providing support through resources for mental health and psychosocial care of staff, including trainings for self-care, would be useful in preventing these outcomes.

So there [are] lots of cases of burn-out and stress and lots of turnover of staff. So there is need for support so staff can be fresh and take on more stories. Otherwise if they continue, they will poison the clients and so I think that is another area of support.

## Discussion

This qualitative study investigated the perceptions of barriers and challenges to implementation of new WHO guidelines for the management of conditions specifically related to stress in Ugandan mental health clinics. Perceptions on the new guidelines were collected from 19 mental health professionals. The study participants were supportive of the guidelines in theory; however, they anticipated a number of practical challenges in guideline implementation, many of which were not distinct from challenges they were experiencing with their current treatment practices. This was true, in particular, for the guidelines on the management of acute stress symptoms and PTSD, in which the recommendations call for an emphasis on psychological interventions over the use of pharmacological management as a first-line treatment. Our study suggests that although there may be widespread support for the idea of guidelines in Uganda and their specific content, in practice, their implementation will meet critical barriers. These include challenges that can be described according to similar levels often defined in dissemination and implementation research: client, provider, organization, and policy [41, 42].

### Client level challenges

Our study suggested that cultural adaptation of guidelines is important at the client level. Participants expressed concern that some of the psychological interventions recommended by the guidelines would not translate to Uganda. They believed that participants may not engage in a treatment that was considered to be fundamentally different from previous experiences at health clinics (e.g., EMDR). In high-income countries, literature suggests that the inclusion of “alternate versions” of guidelines tailored to specific contexts improved guideline use [26]. Similarly, previous research in LMIC has indicated that adaptation of guidelines to fit local contexts and cultures increased the likelihood of successful implementation [43]. Elements of the guidelines that may require adaptation, such as EMDR, should thus be tested and adapted if necessary before they are adopted on a widespread basis. Beyond implementation of the current WHO recommendations, future development of similar guidelines could be greatly strengthened if there was improved knowledge on the effectiveness of existing cultural practices for managing conditions specifically related to stress [17].

An extra level of complexity in Uganda is conferred by the large number of cultures represented by the over 40 ethnic groups and, as a function of conflicts in South Sudan and the Democratic Republic of Congo, the additional cultures of these displaced populations now living in Uganda. Participants reported that while some clients may view EMDR negatively, for example, indeed as “witchcraft,” others who had strong cultural beliefs in traditional healing may be more likely to embrace it. The impact of culture on treatment acceptability may therefore not be consistent across sociocultural groups within Uganda, and this will need to be an additional consideration during implementation efforts.

One cultural belief for many clients in Uganda according to our participants is a very strong expectation for medication when seeking services at a health clinic. This holds for mental health problems as well. Many of our participants expressed skepticism that clients would accept psychological intervention (CBT-T, EMDR, psychological first aid) in the absence of or in place of pharmacological management. Our participants believed that providing increased amounts of psychoeducation to clients in the early stages of intervention would be helpful in improving intervention acceptability and, ultimately, outcomes. A recent study by Prost et al. (2013) found that psychoeducation significantly improved knowledge of mental illness and medication compliance among participants with psychiatric disorders in a Ugandan outpatient clinic. Psychoeducation was not effective in improving attendance at follow-up visits, however [44]. Additional development and testing of the effectiveness of psychoeducation in improving psychological intervention acceptability among clients in LMIC would be helpful in this regard.

Finally, several of our participants discussed the limitations of providing care for persons with conditions specifically related to stress that also have poor social situations or difficult home environments. Their argument was that interventions recommended by the guidelines can only be so effective when clients are experiencing other chronic adversities such as domestic violence, living in refugee camps, high unemployment, or facing land wrangles with family members. Similar difficulties are likely in complex humanitarian crisis settings in other LMIC as well. There is currently mixed evidence in terms of the effectiveness of psychological interventions in settings that have active conflict or are in a current state of emergency relative to post-conflict or post-humanitarian settings [17, 45–47], so more research is needed in this area. Currently, the consensus is that psychological and social interventions should occur concurrently when both types of problems exist [17].

### Provider level challenges

Providers in our study discussed the issue of guideline rigidity, such as the discontinuation of pharmacological management for acute stress disorder, as a barrier to implementation. They were concerned that guideline implementation might result in the care they provide becoming overly regimented. Such guideline rigidity may be inferred from recommendations in the WHO guidelines that have a ‘strong’ recommendation (as opposed to ‘standard’), which indicates that the recommendation should be followed in all or almost all circumstances. ‘Standard’ indicates that there may be cases in which the recommendation does not apply. Many of the study participants believed that the guidelines must be flexible, both in terms of the ability to prescribe medication on a case-by-case basis and also with regard to cultural modification of interventions as needed. In a sense, our participants expressed that their extensive training and experience managing conditions related to stress within the cultural context of Uganda should count for something even if it stands in occasional contradiction to the WHO guidelines. Notably, a recent review of clinical guideline implementation in LMIC found that across 17 included studies, perceived credibility and acceptability of the guidelines by providers was one of the most salient predictors of successful implementation [43]. Furthermore, the adaption of guidelines to fit within local contexts is an essential facilitator of implementation [17, 48]. In other words, the ways in which the guidelines are interpreted and perceived by clinic staff impact the likelihood of adoption and implementation. Dissemination efforts must therefore be clear on the flexibility of guidelines and how this may vary across specific recommendations (e.g., between ‘strong’ and ‘standard’ recommendations).

Implementation research in LMIC also suggests that including higher level providers (e.g., psychiatrists) in implementation strategies often and, crucially, *early* in the process is associated with a greater likelihood of accepting the recommended modifications outlined by the guidelines [43]. Implementation strategies in Uganda and other LMIC should therefore include early engagement and consultation with expert providers in Uganda on the appropriate times in which their clinical judgment (e.g., use of pharmacological management) could be used within the proposed guidelines. The providers could also be consulted on developing methods and indicators for monitoring and evaluating the uptake of and fidelity to the culturally adapted guidelines [43, 49].

### Organizational and policy level challenges

From an organization-level perspective, which includes both leadership and decision-making [41], a critical prerequisite for the success of guideline implementation is dissemination from guideline developers to international non-governmental organizations and subsequent dissemination to staff within those organizations. Notably, only one of our participants (a psychiatrist) was aware of the specific recommendations included in the WHO guidelines. There is currently an effort to widely disseminate the guidelines through the mhGAP-HIG, which is becoming a tool used by many international non-governmental organizations. There are currently no published reports about the effectiveness of the dissemination effort.

One of the most consistently described challenges by our participants was the lack of training in the types of psychological interventions endorsed in the WHO guidelines, specifically CBT-T and EMDR. It is also critical therefore that organizations provide appropriate trainings to their staff in order to increase the likelihood of guideline implementation. This obstacle was anticipated in a previous review that analyzed potential difficulty in implementation of the WHO guidelines in LMIC [17]. Although basic mental health resources do exist in Uganda through the Ministry of Health and non-governmental organizations, the lack of qualified and trained specialists in the WHO-recommended psychological therapies remains a significant barrier to guideline uptake. The guidelines themselves state specifically that these therapies should only be conducted by professionals with the appropriate training.

Naturally, the strategy for overcoming this challenge would be additional trainings on CBT-T and EMDR for mental health staff, and this was the most frequently mentioned strategy for effective implementation of the guidelines in our study. The participants we interviewed were supportive of following the WHO recommendations to use these treatments, but they appropriately asked that they first receive training in how to do them effectively and safely. Many of the participants had received brief trainings in CBT-T, and, although they were able to deliver certain components of the therapy, many seemed hesitant to say that they were confident in their ability to deliver a full course of CBT-T.

The challenges with providing comprehensive trainings in these methods on a wide basis are twofold. First is the obvious limitation of financial resources. Appropriate training on CBT-T and EMDR would be lengthy and require facilitation by expert trainers, both of which would carry significant cost. From the policy level, this problem is compounded by the lack of prioritization for mental health care in most LMIC (including humanitarian settings, post-conflict, and otherwise). On average, Ministries of Health in LMIC devote less than 1 % of their budgets to mental health [50]. In addition to the financial challenge, the effectiveness of short term, theory-driven trainings was brought into question by participants we interviewed who had experience with conducting training sessions in Uganda. That sentiment argues in favor of a larger, longer, more comprehensive specialized training in something like CBT-T or EMDR compared to brief 3- to 5-day general trainings that are currently popular.

There is increasing evidence from LMIC that training lay counselors who have no previous mental health experience in CBT-T or other specialized techniques is feasible when done following an apprenticeship model [51] and that counselors trained in this manner can provide effective psychological treatments under close expert supervision [52, 53]. It is not yet known, however, whether this type of model can be sustained over the long term in LMIC given the supervision requirement (often done with experts in high-income countries) or the extent to which it can be successfully integrated into existing services. This is an important future avenue of investigation for implementation research, and a recent initiative, the Programme for Improving Mental Health Care (PRIME), may be helpful in this regard. PRIME was started in five LMIC, including Uganda, and aims to “generate evidence on the implementation and scaling up of integrated packages of care for priority mental disorders” [54].

The participants in our study described extremely high client/provider ratios (reportedly as high as 75:1 *per day* at outreach sites such as refugee camps by participants in our study) as a barrier to effective care. One participant discussed the inherent difficulty in attempting to manage acute stress disorder with only psychological interventions when those interventions could only be delivered perhaps once or twice in a month due to a lack of qualified staff. Thus, the lack of trained staff and resources to pay trained professionals who are capable of delivering these interventions remains a problem. The lack of trained mental health personnel is common in many other LMIC [50, 55], and the treatment gap between the availability of services and populations in need of services has been estimated to be in excess of 75 % [56]. With such a substantial disparity between need and service provision, even a threefold increase in trained specialists would not alone considerably close the treatment gap. The costs (both in terms of finances and time) associated with training and supervision also suggest that massive increases in highly specialized training alone will not be enough for successful guideline implementation throughout LMIC.

A possible way forward may therefore be a stepped care or task sharing approach [17, 57]. This type of model was mentioned as a potential method for implementation by participants in our study in thinking through strategies for psychological interventions to reach more people. The idea expressed by many participants would include training community mobilizers and village health team members in basic counseling, including stress management, one of the three evidence-based treatments identified in a recent Cochrane review (and also endorsed in the WHO guidelines) [58]. Those with significant or severe symptoms would be referred to the more highly trained mental health professionals, the counselors, and psychiatrists. The training for these higher level providers could take a trans-diagnostic approach [17, 59], given the high rates with which our participants reported managing complex PTSD and other commonly co-occurring disorders. Studies have also indicated that the mental, neurological, and substance use problems experienced by populations in humanitarian settings (including northern Uganda) go beyond the common mental disorders often studied among these populations [6, 60]. Therefore, the providers would need to be trained to detect and appropriately refer cases of epilepsy, psychotic disorders, and alcohol and substance use problems.

In theory, this stepped care method with professionals trained in a variety of treatment approaches would partially reduce the client/provider ratio that is currently one of the major burdens on the mental health-care systems in LMIC. A recent Cochrane review of studies conducted in LMIC found that this type of stepped care approach resulted in superior client mental health outcomes [61]. A study from the PRIME initiative by Mendenhall et al. (2014) found that stepped care was perceived as acceptable and feasible by community members, service providers in primary care centers, and clients [62]. They also found that barriers to successfully implementing a stepped care approach were similar to the challenges identified in our study, including a need for increased human resources and adequate training [62]. In Uganda, determining the degree to which community mobilizers could be trained and supervised on a large scale in delivering *nonspecialized* interventions such as stress management and determining how best to coordinate care between the mobilizers and specialists are questions that should inform future research. Cost effectiveness or cost comparison trials of these types of implementation approaches are also warranted. It is important to note that even with a stepped care approach, support by policy-makers and funding by Ministries of Health is critical for training and hiring specialized mental health staff (for treating clients, and in turn training and supervising community mobilizers and health workers) [62].

### Limitations

This study had several limitations to consider. First, our interviews were limited to current mental health care providers. Although we reached saturation on several themes with these types of providers, we were unable to complete interviews with general health policy-level staff, such as district health officials, or from clients themselves. Input from these types of informants will be critical to successful implementation of the guidelines. Second, we asked participants to provide their opinions on the guidelines immediately after learning about them for the first time. Although we gave the participants as much time to read through the guidelines as they wanted and asked them to respond only when they were ready, it is possible that these initial reflections from the participants may be different than if they had more time to fully digest and think through the recommendations over a longer period.

## Conclusions

The findings from this qualitative study in Uganda confirm earlier suggestions that there will be critical challenges in implementing the WHO guidelines for the management of conditions related to stress in low-resource settings. These include difficulties at client, provider, organization, and policy levels. Based on the findings of our study, we have synthesized key recommendations for guideline implementation research and practice efforts in LMIC.

Research at the client level should include efforts to increase knowledge on the effectiveness of existing cultural practices to improve mental health. Practically, implementation efforts would benefit from the development of a Ugandan version of the mhGAP guidelines, specifically addressing cultural adaptations to psychological interventions while taking into account the various ethnic groups and refugee populations in the country. Research should also focus on evaluating the effectiveness of psychoeducation on improving mental health knowledge and treatment adherence with a particular focus on raising client awareness on the potential effectiveness of psychological compared to pharmacological interventions. Improving knowledge on how to effectively integrate mental health interventions with programs designed to address social, economic, and physical health problems, such as gender-based violence, poverty, and HIV, will be critical to ensure continued mental health treatment benefits. All of these research efforts should ideally be designed to evaluate both clinical and implementation outcomes.

From a provider-level perspective, researchers should prioritize investigating sustainable models of training that include ongoing supervision and support. Longer-term in-depth trainings with providers may increase their ability to administer guideline-recommended psychological interventions compared to briefer 3- to 5-day trainings. Additionally, feedback should be elicited early and often from mental health providers in settings where guidelines are to be implemented and they should be consulted on methods and indicators for monitoring and evaluating the uptake of and fidelity to the culturally adapted guidelines. Finally, given the significant burden of the high provider/patient ratio and the potential stress induced by this workload, a system for monitoring and supporting the psychosocial well-being of providers should be implemented.

Research priorities from an organizational and policy level should first focus on examining the fit of the guidelines with a task-sharing/stepped care approach to mental health service provision. Second, guideline dissemination and implementation should be monitored and evaluated systematically. This is particularly important for dissemination efforts given the lack of previous studies documenting this process and/or barriers associated with it in LMIC. Finally, advocacy efforts must be made to policy-makers for a greater investment in highly trained mental health staff and for increased supervision capacity of current higher level staff (e.g., psychiatrists and psychologists) to address shortage in human resources, possibly using an apprenticeship model approach.

Reviews of studies in both high-income countries and LMIC found that instructions on monitoring guideline use, such as checklists, improved the uptake of and fidelity to new clinical guidelines [26, 43]. Such checklists and guides in combination with the results of this and other qualitative investigations into guideline implementation should be used to inform the development of quantitative tools to systematically measure the implementation of the WHO guidelines in low-resource contexts. The solutions to overcoming guideline implementation barriers offered by participants in our study should be further developed and tested in a rigorous scientific design. Ultimately, the success of implementation will rest on incorporating the input from providers, clients, organizations, and policy-level staff, and we therefore recommend an integrated, active translation approach that includes all of these perspectives in future efforts to implement WHO guidelines for problems related to stress in LMIC.

## Additional files

Additional file 1: Table S2. Contains a table summarizing themes on common types of conditions related to stress that are treated in northern Uganda. (DOCX 13 kb)

Additional file 2: Table S3. Contains a table summarizing themes on current practices for managing conditions related to stress in Uganda. (DOCX 17 kb)

Additional file 3: Table S4. Contains a table summarizing themes on barriers to WHO guideline implementation. (DOCX 22 kb)

Additional file 4: Table S5. Contains a table summarizing themes on strategies for addresssing barriers to guideline implementation. (DOCX 20 kb)

## Abbreviations

CBT-T: cognitive behavioral therapy with a trauma focus
EMDR: eye movement desensitization and reprocessing
LMIC: low and middle-income country
PTSD: post-traumatic stress disorder
WHO: World Health Organization
