# Supplementary material for: Challenges for the implementation of World Health Organization guidelines for acute stress, PTSD, and bereavement: a qualitative study in Uganda
Source: Implement Sci. 2016 Mar 15;11:36. doi: 10.1186/s13012-016-0400-z (PMC4793547; doi:10.1186/s13012-016-0400-z)
Supplement: Supplementary file 1 — Contains a table summarizing themes on common types of conditions related to stress that are treated in northern Uganda. (DOCX 13 kb) [file 13012_2016_400_MOESM1_ESM.docx]

| **Table S2.** Themes related to research objective 1: common types of conditions related to stress that are treated in northern Uganda | | |
| --- | --- | --- |
| **Theme** | **Definition** | **Example** |
| Conflict-related trauma continues to affect mental health, but ongoing adversities are important | Although the civil war ended almost 10 years ago, traumas experienced during that time are still commonly reported by clients, in part because they have not received appropriate treatment in the ensuing years. Many clients also present with recent trauma experiences, commonly domestic violence and “land wrangles.” These recent events sometimes serve to “trigger” previously experienced war-related traumas. | “Most trauma we see is trauma as a result of war. Many people experienced severe loss during the war, they lost someone who was supporting them so much, they lost all of their children… and many went through the experience of abduction.”  “But we also see a lot of on-going trauma—domestic violence, gender-based violence, physical violence, interpersonal violence, that is unrelated to the war. These have distal effect.”  “This is mostly common with ladies and domestic violence. It is common. Because you find maybe she had been initially raped during the war and then the husband beats her, even insults her, tells her ‘I will give you to the rebels.’ So it becomes a real trigger.”  “We have realized that we have new trauma, which is already in place...The new trauma include: one, domestic violence; two, land wrangles, where people are struggling for land because they have been in the camp for some good years and now have returned back home so everybody wants to grab land. |
| PTSD is commonly treated but is often co-occurring with other mental health problems | PTSD is commonly diagnosed and treated by the providers, particularly in rural outreach efforts and visits to refugee camps. They find, however, that the PTSD is often complicated by comorbid mental health problems, both related and unrelated to trauma. | “The mental health problems, especially in the sub county is majority PTSD”  “And for some of them because of these symptoms…they look for all the options to make themselves feel a bit comfortable, especially at night, whereby they start drinking, they start small but then they take more and become dependent on alcohol.”  “The most common problems which go with PTSD both in clinic and outreach is depression. They are almost like brothers and sisters. They move together, that is my observation… If you see a person is having PTSD they also have depression….It is more than 50%.”  “The PTSD cases that come are so complex, complicated with other psychiatric comorbidity.”  “Comorbidity is the norm rather than the exception.” |
| Many non-trauma mental health problems are also common, especially in more urban areas | Although the clinics were designed to specialize and treat PTSD and other conditions related to stress. The providers must frequently manage other mental health conditions unrelated to stress due to the high demand. | “Majority of those who come to this [urban] clinic actually have other types of mental health problems which are not necessarily trauma related…In this place, if we were to take the greatest percentage of persons that we get here, you will find that epilepsy is actually the number one…Then, there is also alcohol-related mental illnesses…Then, other mental illnesses like schizophrenia, like also these other anxiety disorders, a good number have dissociative disorders like conversion disorder, especially female, young school-going age that we receive in this place.” |
|  |  |  |
