# Supplementary material for: Challenges for the implementation of World Health Organization guidelines for acute stress, PTSD, and bereavement: a qualitative study in Uganda
Source: Implement Sci. 2016 Mar 15;11:36. doi: 10.1186/s13012-016-0400-z (PMC4793547; doi:10.1186/s13012-016-0400-z)
Supplement: Supplementary file 2 — Contains a table summarizing themes on current practices for managing conditions related to stress in Uganda. (DOCX 17 kb) [file 13012_2016_400_MOESM2_ESM.docx]

| **Table S3.** Themes related to research objective 1: current practices for treatment of conditions related to stress | | |
| --- | --- | --- |
| **Theme** | **Definition** | **Example** |
| Psychoeducation is a significant component to management of stress-related conditions | The providers all discussed having a “health talk” at the beginning of a clinic day, informing clients in the waiting area of signs and symptoms of common mental disorders. But some went further to note that psychoeducation is actually an important part of the treatment process itself. | “So always our recruitment of patients, we get them through health talk. Like when we go we find the health centers, the first thing we do is health talk…we talk about PTSD, the effects of PTSD, the symptoms, the causes, then later on we go and settle in our position and we find new clients follow us.”  “I also think that a strong component of what we do is a psychoeducation approach. Which is that a lot of people know they have gone through trauma but they cannot link it to the symptoms or the mental health problems that they are presenting with. What we are doing in the clinic most of the time is providing psychoeducation to link the trauma to the symptoms and hopefully it helps both. Dealing with the trauma and also relieving the signs and symptoms of trauma.”  “Their needs are psychoeducation. The person that says, ‘you need to use a trauma-focused approach, a non-trauma-focused approach.’ Be the person that explains, ‘it is not witchcraft it is not bewitchment, it is not spirits coming back to you. It is flashbacks and flashbacks happen in post-traumatic stress disorder.’ So oftentimes I think what patients need is to explain the linkages of the trauma, their symptoms, and how they can deal with the symptoms themselves. If that doesn’t work, if psychoeducation doesn’t work at the outset, then you get more focused approaches.” |
| Assessment is done in a holistic manner | Across sites and position (e.g., counselors and nurses), the providers consistently described a holistic approach to client assessment and creating treatment plans. Social workers, for example can address problems that psychiatrists cannot, such as domestic violence. | “I would say… we have a holistic approach, while the psychologist, the social worker is there, the clinical officers, the patient, we see the patient from all areas which I think helps with their recovery.”  “So we work as a team because we want to handle patients in holistic manner. Because when you do the clinical assessment, if you identify social problems that is when you refer to the social worker to do her part. When you also identify psychological problems, psychological symptoms, that is best treated by the counselor. So that is how patients are handled.” |
| Patients with severe symptoms are often given medication before attempting psychological treatments | Providers described a deliberative process through which medications are prescribed to those presenting with trauma symptoms, however, it was generally thought that this should be done for clients with severe symptoms before psychotherapies or other psychological treatments were tried. There were differences across provider type, however, with this practice more often supported by psychiatrists as compared to counselors. | “I think…it [counseling] is very small. It is not really paid attention to, it is all about diagnosis and what kind of medication… It [medication given before referral to a counselor] happens. But then if it gets to me and I discover so many things, that the drug is not needed I get back to the clinician and we discuss.”  “If we find out that this patient has very high level of symptoms, we don’t always encourage them to start psychotherapy. What we do first is start them with medication because the aim is to first reduce the symptoms, if the symptoms are reduced that is when we enroll them for psychotherapy, the talking therapy.”  “For this kind of sleep problem, we really have not been prescribing drugs, because the fact is the benzodiazepines are addictive drugs. And if they come in and say they have been using we try to discourage. We emphasize to help people to come out of the entire trauma situation. When somebody begins to come out of that their sleep will improve also. So we have not been giving them drugs for that” |
| Group therapy is commonly used and preferred over individual therapy | Group therapy is thought to be effective and widely used (vs. individual therapy) due to high demand and its ability to treat several clients simultaneously. Individual therapy is used if a client is severe or refuses group therapy. | “We do group support psychotherapy to counsel clients who have similar presentations so that they can encourage each other and get from one another how one is coping up.”  “Actually in most cases, let me say 90%, it is OK with them to be in a group, knowing they have gone through similar experiences so they don’t reject. And those who we realize they are not coping as much in the group sessions, we still continue handling them in the individual sessions. There are those who cannot express themselves so much, so we single them out… we still continue with them individually to see they are helped very well from the problems they went through.” |
| There are myriad challenges to current treatment approach | The providers believe their treatments are generally effective at reducing trauma symptoms but that there were challenges to the current treatment approach and strategies for overcoming those challenges. | What I always think about, in order for our team to improve, which is a little bit challenging, is being a specialist in each kind of thing you do. Like, if we could have a psychologist who specialized in CBT, maybe two or three, that always did CBT in detail. I am a nurse, so I could only really specialize in my area of assessment, assessment, assessment, and medication. That is the cap I always see. Because you see, I am currently 3 in 1. I do clinical, I do counseling, and sometimes, I feel tired.  “We have a big gap in psychotherapy…Clinicians do not have that much time to attend to patients compared to the counselors. And therefore they tend to miss out a lot…from patients. And then when the patients come to counselors, you really discover so many things that should have been captured by the clinicians and this brings us back and forth, because if I discovered something that needs attention by the clinician I need to go back to the clinician that saw the client first. I would say that also that leads to misdiagnosis sometimes, because you missed a lot or you were not giving time to the client to narrate all of the concerns that they came with to the hospital... But if the clinicians were equipped with the skills for talking therapy they wouldn’t have missed, they wouldn’t have misdiagnosed, they would have given the right diagnosis.”  “We don’t have the resources, we rely on what we have. It is what [clinics] can afford, which is going to each outreach site twice a month. But really the concern you have put is really, really serious, you really find this patient needs to meet twice this week maybe or every week once…it is a huge challenge”  “The reason being, we sometimes have patients who really need medication before the psychotherapy, but sometimes to our surprise because this is a ministry of health team, we go there and there is no medication…Because there are some patients who you really see they really need drugs but the drugs are not there.” |
| Populations have different treatment needs | Types of mental health problems and needs of populations differed based on location (refugee settings vs. clinics in towns). | “There is a lot of difference because the people in the camp, because of the state they are in, of course their beddings are not OK, and even the house itself is not really fine like people here [in town]. But we encourage them, however state or condition this room may be, but trying to make it clean to yourself so you feel you are in a safe place and all that. I want to give a scenario, there was a colleague, she was a wife to some army officer in Congo, and the husband was killed and she was here in the camp, they actually killed the husband and she was raped, and the husband was killed so she just could not sleep. And she was sleeping on carpets. So we were trying to bring this sleep hygiene. There is a lot of difference between people in the camp and in the nicer area in town here. So it is hard.”  “It is common for us to see depression comorbid with domestic violence. For example, in the outreach site, whereas here [static clinic] you see more somatization and depression” |
