# Supplementary material for: Challenges for the implementation of World Health Organization guidelines for acute stress, PTSD, and bereavement: a qualitative study in Uganda
Source: Implement Sci. 2016 Mar 15;11:36. doi: 10.1186/s13012-016-0400-z (PMC4793547; doi:10.1186/s13012-016-0400-z)
Supplement: Supplementary file 3 — Contains a table summarizing themes on barriers to WHO guideline implementation. (DOCX 22 kb) [file 13012_2016_400_MOESM3_ESM.docx]

| **Table S4.** Themes related to research objective 2: perceptions of barriers and challenges to implementing the WHO guidelines | | |
| --- | --- | --- |
| **Theme** | **Definition** | **Example** |
| Qualification of staff and the need for additional training | Although, CBT-T was thought to be effective, providers emphasized the need for staff to receive training in order to appropriately carry out this intervention | “Because they come up with very beautiful guidelines like this, but then if you find the training we don’t, we have more clinicians in the mental health section than psychotherapists. And then it is very, very difficult to implement these guidelines and have the majority of people in this section to have a background of medication.”  “Yes, CBT, but also another challenge is we need some of these trainings. People are also forgetting and you need to update yourself. Like me looking at this guideline, me I didn’t know something like these things existed.”  “What if there is no expertise to do CBT and EMDR? We know that SSRIs, the antidepressants have a small effect size, that we know. But we also lack the expertise for these two therapeutic techniques. So we find ourselves having to describe some antidepressants, perhaps sometimes for placebo, but we do prescribe it.” |
| Patient/provider ratio is too high | Many providers mentioned the reality that their client loads are simply too high to effectively manage, especially if psychological (vs. pharmacological) interventions are prioritized. Many described feeling overburdened and that burn-out among health workers was a possible outcome. | “Then also, if the guidelines are to be appropriate I encourage the government and Ministry to make sure they recruit enough manpower, because individual therapies take time. So when you see people with those symptoms and you told them you are not giving them drugs but just individual counseling sessions, you know you are going to take time. Not less than 30 minutes per patient. So how many patients are you going to see in a day if they are 75 and you are 3 people? Can you?”  “It is a challenge that we face: how do you do mass therapy for massively traumatized communities as we see in mass trauma in northern Uganda? It is a question I was asked by a professor when I was a medical student, what mass therapy can we give to our people who have been massively traumatized? I didn’t have an answer. I still don’t have an answer.”  “I think I need to add there is a lot of stress in our area, especially among health workers. Why? Because during that period of the war, all the victims are people who were wounded, were brought to the facilities and the health workers were dealing with all these kinds of things going on. So you will find nowadays if you move around, you find lots of health workers have problems, post-traumatic stress, they have that, they have this. And people do complain that they are going through that stress. So I think when they implement this thing, they should first start with the health workers, then go down.” |
| Providing consistent, frequent treatment to clients | In addition to difficulty in treating clients due to a lack of trained professionals, providers also discussed that reaching clients in rural areas and refugee camps was a challenge and resulted in irregular therapy schedules, sometimes as little as once a month. | “You know that there is a recommended frequency for conducting psychological interventions, for implementing psychological interventions for having a desired impact. When you are in a month going twice, you may not achieve well. For some outreach stations we only go once a month. It is causing trouble, you can’t run this group for 8 months, I mean! First of all, in one month time after 30 days they have forgotten what you have discussed. Remember, in these communities, they do not write, they do not read. So much of the thing is done just talking. And when you talk and you come after 30 days, they have forgotten everything.”  “So we realized that on the outreach days we were overwhelmed with the number of patients and the patients were not improving markedly. Because remember talking to a patient today and then next month. There is problem between there. Now, even in this twice a month visit, though the work load has reduced we still have that problem. But it is challenging because there are some patients who should be attending psychotherapy even weekly. But our hands are tied because of the funding. So that is a challenge. But medication, we can give for the two weeks, no problem. It is easy. But the problem is the psychotherapy.”  “You know that there is a recommended frequency for conducting psychological interventions, for implementing psychological interventions for having a desired impact. When you are in a month going twice, you may not achieve well. For some outreach stations we only go once a month.” |
| The use of medication: clients have a strong expectation of receiving it needs/wants | Many providers discussed the challenge of clients arriving at the clinic with a preordained expectation of receiving medication and that being offered psychological treatment instead was in many cases not acceptable. This leads to clients dropping out of treatment. | “The only challenge is that in our setup here people mostly believe things like medication are helpful. When a patient comes to you, you just talk to them and then don’t give them any drugs to swallow or to be injected, at times they may feel as if you have not helped them.”  “In most cases, patients in our country coming from home to the hospital, the first thing they expect is drugs, and if they don’t get drugs it is like they have gotten nothing.”  “Now for us if you say those ones [medications] are not recommended, then people will start running away from us. Now what we should be doing, tactics, maybe say here is one injection that will help your problems, and from there we do the psychoeducation, but now you if you start with psychoeducation without giving the medication nobody is going to come back. Nobody! Nobody will come back! Because they will go to a place where they can dispense drugs from the counter. They will just go there.”  “If an African comes to you in the clinic or hospital and you don’t give medication, [snaps fingers], what will happen is very, this same man or woman to come to you next time, because they believe you have not worked, you have not given medication, you have not worked…So when they come here and you don’t give them medication [snaps fingers], that is the end. Somebody may not come back to the hospital. So now go back to the traditional healer, the local hubs, the witch doctor…In most cases because they feel it is the drug which is going to remove or reduce the physical symptoms they are having, not the psychotherapy.” |
| The use of medication: many providers thought it was necessary for clients with severe symptoms. | Many providers felt that medication was necessary for clients with severe symptoms, particularly those with comorbid mental health problems. Following a first-line medicine treatment, many thought that psychological treatment could follow. | “What is the degree of the symptoms? If these are people with symptoms are too high, not sleeping at night, all the memories are there, not sleeping, nightmares. Eh. It becomes tricky to start using CBT alone without first helping this person with a few antidepressants to bring down the symptoms so you can bring out the aspects of psychological interventions.”  “I would say that there are some symptoms that talking therapy alone would not remove. Like re-experiencing. There are some symptoms like the one with burning sensation, sometimes I feel like they cannot be without medication.  “OK, so sometimes people come and they are having these acute stress issues and it is like they are, it is like even to start to talk to them and work on their cognition may not really come, so I think some medication in the beginning before they start the cognitive behavior therapy. The CBT is good, but I think in some, especially in acute stress, someone needs his mind to first come together using medication.”  “Medication is trial and error. If you have severe depression, doesn’t matter if it is related to trauma or not, I treat that depression with antidepressants. If you need them, you need them. To deal with the biological symptoms and of course we talk about the psychological symptoms that will not usually be amenable to medication only. I think it is good to have guidelines but I think some of these guidelines are too trigger happy. There is a role for medication.”  “Well, that goes back to the old arguments: psychotherapy alone, medications alone, medication and psychotherapy. To me, I still go with the old teaching: medication and psychotherapy works best. It is very difficult to treat some of these disorders when you have these symptoms and waiting for time for psychological recovery.” |
| Cultural acceptability of the interventions | Providers felt that some of the guidelines may be challenged by the cultural context of Uganda. For example, many cited EMDR as an inappropriate intervention culturally. | “It is well put here that culturally appropriate. Sometimes these cultural issues interfere with our work. Like most of the PTSD symptoms, it is somehow fitted spiritually. In our culture there is a lot of spiritual belief, so you find someone who has PTSD complex, and the relatives sometimes if you don’t really watch them well, or talk to them well, you find the relatives are doing both, they go to an appropriate cultural practice at the same times they come to the hospital. And so cultural sometimes it is a challenge.”  “Yes, I would say the challenge is the cultural norms, cultural understanding. Sometimes when clients come and they come with their caregivers, there is this cultural perspective that has been going on all this while. They will say this person has been bewitched, maybe they had a conflict at home, or they will say this person is sending it to you. These are the challenges we face.”  “I see a challenge with eye movement desensitization and reprocessing. To me, this eye movement cannot be effective in our community. They are not going to understand this. In our community, we have our beliefs, we have the traditional beliefs. ‘At least, I would accept CBT.’ We know CBT is good and wide enough, it has all the aspects of cognitive restructuring, psychoeducation, reassurances, all of those other things. That can be very helpful for us. Otherwise, some of these non-medication treatments can hardly apply in the community. Like EMDR, no. No.” |
| Poor social, health, and economic situations | Providers pointed out that even if psychological interventions are effective and a client improves, oftentimes they are living in poor, or sometimes, abusive situations, have serious physical health problems with low access to care, or difficulty finding work. | “Treating PTSD, it is the most frustrating thing to do. One of the most frustrating things to do. Because treating PTSD you treat, and you need to be like, if you are treating a patient, you can’t only do the clinical part of it and leave the livelihood, the social-- that is the most important part of it. So if you treat the PTSD, and at the end that person is having a social intervention that is good and good position socially they will be the happiest. But if the social aspect of it is not handled well, there is the probability of relapse.”  “Many times after seeing these patients our challenge is, still there will be other social problems that come in. so first we need to help this person. Because they always need somebody’s support.”  “The other challenge, social challenge, when you talk about social interventions. People think of tangible things. ‘So now you have treated me, I am better, so what next? I lost my cattle, I lost this and that, my property. So it is good you have come you have helped me I am psychologically better, but what next?’ The social package for them is very much a challenge.”  “Like with these children, they are really so vulnerable. You get a child, a family head is 10 years, then 8 and 6, so they are constructed a small hut and they don’t have food, or uniform, and so you feel what can be done for these children? If there were some kinds of supports for those groups of people. Their basic problems remain, you can provide counseling, but there is something pressing them that is not solved. As much as you can link them to the services in the camps in so on, they often don’t get the support.” |
| Structural barriers to implementation | Structural barriers to guideline implementation included resource limitations (non-human resource limitations), medication availability, management/communication problems, relationships with local community leaders, and transport issues (difficulty in reaching clinics among clients who live far away). | “Medications are not really available. Because if you go to the health centers where the refugees are…oral drugs are not there... Also if the drugs do come, they travel all the way from the camps to the hospital, which is very far and you have transport costs.”  “There is that gap. What do I mean? Right now there is a very big concern about alcoholism…They wanted to come out with some rules and laws and talk about alcohol. But they haven’t even called us, the experts, what will be done, these people are drinking like that, what should be done? They should also inform us so we prepare for receiving those people. They are not like that. That is also a very big gap.” |
