# Supplementary material for: Challenges for the implementation of World Health Organization guidelines for acute stress, PTSD, and bereavement: a qualitative study in Uganda
Source: Implement Sci. 2016 Mar 15;11:36. doi: 10.1186/s13012-016-0400-z (PMC4793547; doi:10.1186/s13012-016-0400-z)
Supplement: Supplementary file 4 — Contains a table summarizing themes on strategies for addresssing barriers to guideline implementation. (DOCX 20 kb) [file 13012_2016_400_MOESM4_ESM.docx]

| **Table S5.** Themes related to research objective 3: strategies for overcoming barriers to guideline implementation | | |
| --- | --- | --- |
| **Theme** | **Definition** | **Example** |
| Training and capacity building for current mental health staff | Providers agreed generally with many of the recommendations but believed that in order to implement them effectively, they would require additional training, particularly in specific psychological techniques. | “I think EMDR, we could also need training on that. And I still believe that we needed also more elaborate training on CBT. Those are the areas we need more training.”  “In our government system, we have a big gap in psychotherapy. There are few counselors who come into the mental health section because with clinicians, clinicians do not have that much time to attend to patients compared to the counselors. And therefore they tend to miss out a lot...But if the clinicians were equipped with the skills for talking therapy they wouldn’t have missed, they wouldn’t have misdiagnosed, they would have given the right diagnosis.”  “These other recommendations most of them are going to work. But before, I recommend trainings. Refresher courses, continuous medical education for the health workers for it to work well. Because in the settings here we know, our existing education, there are some people like my level have missed very many things here.”  “What I have seen is that the counselor or psychologist, if they are lucky, they have been exposed to CBT. But maybe they have not been exposed to another intervention like EMDR. And sometimes these can be working together in combination depending on how the patient is. If, for example, you want to avoid using medication and want to first employ EMDR, you can subsequently employ CBT. But again, the capacity is not there. Like now for EMDR in Uganda, there are very few people…So there is a capacity gap.” |
| Additional staff | In addition to more training, providers felt that in order to provide psychological intervention to large numbers of people, a greater number of trained staff would be required. | “I would say that my recommendation that I would give goes back to more counselors to be in place in order to really, if we had counselors who would go for outreaches. Because now…they have got one clinician who is a psychiatric nurse who moves around to the health center to check on drugs and what is lacking. So it is all about drugs, drugs, drugs. If at least there was a counselor who would move around that would make a big difference.”  “The good thing now for one of our outreach sites, they want to recruit another social worker. At least something good about that outreach site is that we have a female community mobilizer who was really helping when I am running maybe out for the home visit, she can take over. But even better still with the social worker they are going to recruit. But the problem I have is now that one of the outreach sites we are the only women human resources when I go there and the rest are male. So if I am rushing, I have to first finish with the women before going for the home visits because I cannot give this to men community mobilizers because there are things they won’t be free to share with them. So that is a challenge. So for me, to have another female human resource, community mobilizer, that one would help.”  “I don’t know. The solution is more than what we have right now. It has to do with the resources you have and the level of personnel. If it is a primary healthcare giver, then it would be easier for him if he had professionals and he said I think this person needs to see you. But he is everything, he is primary healthcare provider for every disease. So that becomes the biggest challenge. But maybe as you said maybe scale up by training more basic providers who have those limitations we talked about.” |
| Resource allocation/sharing | A number of providers discussed the concept of improved resource allocation and task sharing, including the idea that some services could be provided by lay people, volunteers, and community mobilizers and that it would be possible to train these individuals in more basic forms of counseling. | “Usually we are two facilitators, so if we trained a VHT [village health team] or some other community social worker who is based in the outreach, we would need another person to be with her. I think that is an idea, having another person purposely dedicated to go into the field and implement and run. I think that is, that can work for sure. It can. And it would help. Because much of the work is at the outreach. Much of the work is at the outreach, we see very few patients here. Today it is maybe two patients. But when you are at the outreach you see hundreds of patients. And that is where our impact is most needed. So if we did that it would be better. Because, as the guidelines are saying you need more psychological interventions, but you know how can you have psychological interventions with 75 people? Psychological interventions are something which needs time, if you are going to intervene psychologically, you need a long time with that person to have an impact. There are 75 people [at the outreach].”  “Stress management has a level of at least it can work in my view. If someone is severely, severely affected, how will stress management help a person to recover? So for some yes. But if you look at the continuum of symptomatology, maybe if the person is stressed and don’t have severe symptoms of PTSD like intrusive memories, and dissociating, that kind of stuff, that that kind of person can probably benefit from stress management. So I think we need to build capacity to prove all. But it depends on peoples’ background. They need foundational background in psychology.”  “Yeah, as I said it is a continuum. You need help at every service level. So in the community if you have people trained in stress management, they can do some work at that level. But then you also need to have a service in my view of those who might no benefit or whose cases are more complicated than the community person trained in stress management can help.”  “A stepped care, that is what I am thinking. So you have very few people who would need EMDR, psychiatric care. So for every program in my view you have to have those steps. And so you have family, community, provider level basic, and then you have more advanced care which has very few people and has a lot of challenges.” |
| Flexibility | Many providers felt the guidelines could be useful as a framework within which they had the ability to provide their own clinical judgment. Many believed that a provision for not using medication for acute stress disorder, for example, would be too rigid and would not be an acceptable guideline to many caregivers. | “You know these guidelines, most of them we are doing, except they are just preventing us from doing a few things, especially medication. Yeah, most of them are true. But for depression, for severe PTSD I think some of them we need medication.”  “Protocols sometimes, first of all they can never fit in every context. The medications you have written there in mhGAP are not available in certain contexts, and that is the flexibility. So it starts with trained people who have options that may not be there. So the protocol should be a flexible document; that is by design.”  “There needs to be flexibility. I have seen people become totally psychotic as a result of trauma. So you have to relax the rules a little bit. There has got to be some medication. But not routine. Case by case… It has to be exceptional cases….But, again, you can’t put a hard and fast rule. I have seen people get terror nightmares and bad dreams and if you are sleep deprived for a long time you can become psychotic. I have seen that. And again, not all cases, but case by case. Not routine…Flexible. Trauma therapy, whatever kind, you have to be flexible.” |
| Cultural adaptation | Some providers believed that in order for the guidelines to be effective, cultural adaptations of some of the interventions is warranted. | “The training [should be] be given according to our settings because there are some settings where we may not be able to detect whether this setting can even fit into our cultural practice or not.”  “Then the cultural perspectives, particularly with the refugees are different culture with the providers, even if the provider was nearer one culture of the refugee, the refugee also, there are many, many ethnic groups within Sudan, so that challenge will be there. So the more service providers interact with them, the more they will understand the cultural practice. This must be based on hands-on, on the job, interacting with other cultures.”  “Possession states here are very useful in the treatment of dissociations and conversion disorder. I don’t know if you have witnessed possession states, a ritual washing, the middle of the night, the whole family, going into trance states. When they come out of that, things are gone. So that, I would say, not very properly researched in the Western sense, but culturally useful here. And that is when we argue that traditional healers vs. western trained therapies, who don’t know these things. I don’t know them. But I know they work…Someone did a lot of stuff in Western Uganda about the use of possession states on psychotrauma where patients were presenting with dissociations and somatizations. And they worked. So, few studies, but dramatic results. How best to teach it is something that should be subject to research.  “EMDR? In Africa? I have not seen it work…. The idea of eye movement desensitization and reprocessing or exposure therapy for traumatized people in our communities is a very cultural area. Now, if it was combined with some other traditional accepted method of trauma relief, than yes. Traditional healers use all kinds of psychodramatic treatments. So if it was combined with those, maybe.” |
| Additional psychoeducation to clients | Although psychoeducation is often frequently used already in management of conditions related to stress, many providers feel it should have an enhanced role in educating clients about the effectiveness of psychological treatments. | “I think what we just need to do is to try to continue sensitizing about how psychotherapy works. There is need to explain that to people so that they really understand that this is something that can help them. Without understanding that would leave doubt that they needed some medicines to swallow, that kind of thing, but if they are helped to understand how this talking therapy works, the will come to believe it.: Yes it is very tough. And I would say I could use some help in learning how to convince them.”  “Thinking, still we need to have people more sensitized to trauma because not all know about trauma, it is there, but integrating it in care, the trauma with caregivers would be good.”  “Again, I think that is also one other challenge and it is based on cultural perception of who the doctor is. And it could also be the same challenge with EMDR for example…And then of course to say that people might believe more in medication, whether it is a placebo. But it depends on the psychoeducation provided to the patient when they come. If they don’t have a background and you don’t psychoeducate them before you recommend them to EMDR sessions then they might not believe they are going to get well without medication. Again, we need to gather evidence, but if we explain to them that this is what is happening to you because of this, and this is how the brain might react, and these are the symptoms you might get. And in order to get well maybe this is what needs to be done. Maybe. But I don’t know what you could o but that is my hypothesis.” |
| Staff psychosocial well-being | Participants described the need for staff support as a first step in improving the treatment system. | “So there [are] lots of cases of burn-out and stress and lots of turnover of staff. So there is need for support so staff can be fresh and take on more stories. Otherwise if they continue, they will poison the clients and, so, I think that is another area of support” |
| Group therapy | Acknowledging the limited number of providers and the large unmet need for services, participants described utilizing group, as opposed to individual, therapy to manage the high demand. | “We have revised the means of doing it. They will not be all seen individually, that is how the concept of groups came in. People with similar conditions and similar symptoms will be in the same group.”  “How are we supposed to do stress management or CBT with all these people? Groups” |
